# Lecture Delivered by Dr. J. Brown, before the Macon County Medical Society, January 1, 1867

**Published:** 1867-03

**Authors:** 


### CHICAGO MEDICAL EXAMINER.

N. S. DAVIS, M.D., EDITOR.

VOL. VIII. MARCH, 1867. NO. 3.

### Original Contributions.

ARTICLE XIV.

### LECTURE DELIVERED BY DR. J. BROWN, BEFORE THE MACON COUNTY MEDICAL SOCIETY, JANUARY 1, 1867.

[After the delivery of the Lecture, a resolution was passed by the Society to furnish a copy to the city papers, and both the Medical Journals in Chicago.

MR. PRESIDENT, LADIES AND GENTLEMEN:—

At a recent meeting of the Macon County Medical Society, a resolution was passed requiring the President to deliver a valedictory address before them, and that the public should be invited to attend. In accordance with that resolution I appear before you to discharge that duty. I am well aware, that to accomplish this in such a manner that it shall be both interesting and profitable to this audience, is no ordinary task, especially by one who is not in the habit of speaking in public.

The question is frequently asked, "What is the object of all these Medical Organizations; and what good is to result from them to the Medical profession, or to the general public?" I will endeavor to answer these questions as briefly as possible.

The grand and principal object of the formation of the American Medical Association, was to improve the system of medical education in the United States. Previous to the formation of this Association, there was hardly a medical school in

America whose course of lectures exceeded from twelve to sixteen weeks. Many of them afforded scarcely any facilities to the student of medicine for acquiring a thorough medical education. Almost any illiterate, ignorant, or immoral young man, however destitute he might be of all the necessary qualifications to make a good physician, could easily gain access to some of these so-called medical schools, and after attending two partial courses of lectures he comes out a full-fledged doctor of medicine, to trifle with human life, and in hundreds, and even thousands, of instances during his long and unprofitable life, he becomes the murderer of his own friends and patrons.

The founders of this noble organization, were, many of them, the brightest stars of the medical profession in the United States. Great, learned, and good men—true philanthropists in the broadest sense of the term—and now behold the result of their glorious efforts, and the wonderful reformation that has taken place in American medical schools during the last twenty-five years.

In almost every State in the Union there are one or more medical schools whose professors are men of superior talent and moral worth, and will compare favorably with any of the medical faculties of the old world.

At the present day, a course of medical lectures extends to five months, in nearly all the American schools. The medical student now not only enjoys all the advantages of superior instruction; but nearly every school possesses ample facilities for dissection, large and valuable libraries, costly and magnificent anatomical museums, as well as large and commodious hospitals, with their hundreds and thousands of patients, who are afflicted with every variety of disease that can be conceived of, to all of which the student of to-day has free access. And no young man can avail himself of these innumerable advantages, unless he has gone through a very thorough course of study, under the instruction of some first-class physician. Must be a young man of the best of morals; must possess a good English education, to say the least, and he must have a certificate to this effect from his preceptor. And even this is only a partial enumeration of the great benefits resulting from the united effort of the master minds of our noble profession. This bright constellation of intellects, composed as it is of delegates from almost every State in the Republic, meet annually and discuss the great medical questions of the age, and the vast improvements in medicine, surgery, and all their kindred branches. The proceedings of the Association, including nearly all the discussions of any value, are published in the various medical journals of the country, and by this means nearly every physician, from the Atlantic to the Pacific, from the Lakes to the Gulf, may keep himself thoroughly informed in reference to all the great improvements in his profession.

The success of this great enterprise, thus far, has surpassed the most sanguine anticipations of its most ardent friends.

Next in order are the medical organizations of the various States. Each bears the same relation to the American organization that each State does to the General Government, while every State Association is composed of delegates from the various county and city societies, each and every one contributing to the general good and prosperity of the State Association.

Our own beloved State of Illinois is not behind any other sister State in promoting the great interests of medical education, and in elevating the character, general intelligence, and dignity of its physicans. Have we not the Rush Medical School, as well as the Chicago Medical College? Can we not boast of the profound erudition and world-wide fame of a BRAINARD and DAVIS, who were the founders of these two splendid schools? And we may also feel proud of the St. Louis Medical School, at whose head stands a Pope, whose fame as a surgeon is not surpassed by any on this Continent. Show me a graduate of either one of these schools, and I will show you a splendid practitioner of medicine.

A few remarks now in reference to the Macon County Medical Society.—This Society was formed in 1853, by a few of the most enterprising and skilful physicians who then lived in Decatur. After having formed a constitution and by-laws, by which they were to be governed in all their business transactions, they adopted a code of ethics, which was in substance the same as that adopted by the American Medical Association. This code of ethics is designed as a standard of fixed principles of right and justice in the profession, and the conduct of every true physician is regulated by it in all his business and social relations with his brethren in the profession.

The objects and aims of this association are to mutually improve its members in everything pertaining to medicine, as well as to promote harmony and brotherly feeling in the profession: both of which they are eminently calculated to produce.

From the first day of its organization, some one or more of its members have been required to read an original article on some subject connected with medicine or surgery. The merits and demerits of the paper are discussed and very thoroughly ventilated by the society, nearly all of its members taking an active part in the discussion of the article in all its bearings. To get up a paper of this character, that shall do credit to the author, and at the same time benefit his hearers, is a greater task than might be at first supposed.—No egregious blunders or false principles can be enunciated in the presence of this body of critics. As a natural consequence, a spirit of investigation is at once excited, not only in the mind of the writer, but in that of every member of the Society. Old and new books, as well as the various medical journals of the day, are reëxamined with extreme care to ascertain, if possible, everything that is known in reference to the disease under consideration, with all its symptoms, pathology, and treatment,—and I must say in all sincerity, and without the least intention of flattering any brother physician, that very many papers have been read in the Macon County Medical Society, that would reflect credit and honor to the medical profession.

The harmony and good feeling which have so long existed among your physicians in Decatur, are universally understood and commented upon by all classes of society, and this can be attributed in a very great measure to our frequent meetings.

I have now given you a hasty sketch of the objects and aims of these various Medical Associations. Every neighborhood, hamlet, town, and city, throughout this broad land, are reaping the benefits resulting from the combined efforts of the medical profession in this great work. Is this not so? Are you not especially and very deeply interested in the character and qualifications of the physicians of this city and county? Most assuredly. Is it a matter of no importance into whose hands you place the life of your sweet babe, your lovely daughter, your noble boy, your beloved wife or husband, all that is nearest and dearest to you on earth? There is, in fact, no question of more vital importance to the interests and happiness of a family and their friends, than that of selecting a family physician. He should not only be a man thoroughly informed in all the various branches of his profession, should possess a discriminating mind, a sound judgment, but he should be a man of refinement and general intelligence, of the most irreproachable moral character: in a word, he should be like Cæsar's wife, above suspicion.

I am fully aware that many very intelligent persons upon all other subjects, exercise but little discretion and judgment in this very important matter.

If a young man comes to Decatur for the purpose of exercising his vocation as a teacher, what are the first steps he has to take before he can gain employment? Why, he has first to satisfy the county school commissioner that his moral character is unquestionable. Before he can teach our children the alphabet or the multiplication table, he must undergo a thorough examination in all the branches he proposes to teach.

But how is it frequently in the selection of a physician? One of these itinerant mountebanks comes to Decatur, his flaming handbills precede him for weeks. No one has any knowledge of who he is, or from whence he comes; and with his infamous misrepresentations and base falsehoods he imposes upon the credulity of the ignorant, as well as many intelligent persons. No one of his patrons takes the least trouble to ascertain anything in reference to his character as a man, or his qualifications as a physician, or whether he has ever been inside of a medical school, or whether he has the least authority to practice medicine, except the license which is granted him by the city council.—Such men, ninety-nine times out of a hundred, are fit subjects for the penitentiary, and if they had their just deserts would be consigned to those comfortable quarters, pecking stone for their daily bread.

Professional brethren, I fear that we are not always true to ourselves. Have each and all of us carried out in good faith that sacred obligation which we entered into in refcrence to the prompt collection of our well-earned fees. I fear we have not. And why is it? Is it because physicians, as a class, can live cheaper, or, as a class, have no aspirations to live independently, and to enjoy the comforts and luxuries of life, equal to those who occupy the same position in society? No, my friends, those are not the reasons. The only true reason that can be assigned, on the face of the earth, for this dereliction of duty on the part of the Medical profession of this county, is sheer negligence; a species of carelessness; a miserable and intolerable habit of which we, as a class, are alone guilty.

In our intercourse and business transactions with all other men of this city and its surroundings, we find none of this cowardly and sycophantic manner exhibited in collecting their just dues, from all patrons, and consequently it is expected by the community at large, that no credit can be obtained of the mechanics, grocery men, dry goods, or clothing merchants. In fact, no class of men do a general credit business, except physicians.

Now is it not strange, gentlemen, that the medical profession of even our own town, do not possess sufficient firmness and decision of character to carry out a noble resolution, when the comfort, happiness, and prosperity of their families and themselves depend upon it. Look at the nerve exhibited by proprietors and clerks of business houses in Decatur, in refusing even their best customers credit, for a few days. They will say, no, we have quit that style of doing business. If you have not the money with you just now, we will ticket it to you, and drop it into the change drawer, and you can hand it to us the next time you come in. And what is the consequence if this ticket is not cancelled in a few days? A younger clerk calls upon the debtor, and says, we would like to have that little bill, sir. And if it is not paid forthwith, a constable, in a few weeks at longest, taps you on the shoulder, and says, I have a summons for you. And if the amount is paid then to the sheriff, without any further trouble, does that individual, or any member of his family, get any more credit at that house? No, indeed, my friends. There is nothing wrong about this. It is all right, and you will unite with me in saying "amen" to this mode of doing business. And woe be unto Decatur and its bright future prospects, whenever that day comes that a general credit system is again established.

Now, on the contrary, what is the course pursued by our honorable body, as business men, in this respect? In the first place, we give our whole time and best energies of mind and body, both night and day, to our patrons, not even excepting the Holy Sabbath, that glorious day of rest, with all its joys and comforts, which brings repose to all other classes of men, and even to the brute creation, but comes not as a day of rest to the weary and care-worn physician. But on the contrary, with all the grave responsibility of very many precious lives entrusted to his skill, he moves on his endless and never ending round, visiting the sick and dying at all hours, always with a smile and a sympathetic word of encouragement, administering some God-given remedy, which cools the parched tongue of fever, and lulls the pain distracted body as by some magic spell. He is frequently exposed to the hottest summer's sun, or the rain in torrents, or to the frosty elements of cold and dismal wintry nights. After doing all of this, which is but a faint picture of the hardships experienced by every true physician, do our friends compensate us as promptly as they do other men who work for them? No, indeed, gentlemen. We return weary and fatigued over long and rough roads, through mud and sleet, at the hours of one, two, and three o'clock in the morning, broken of rest, to dream the remainder of the night of the beauties and glories of the medical profession, almost feeling in our heart of hearts, that the messenger who had dragged us forth at the hour of midnight, from our comfortable beds, had conferred a great favor upon us. And we carry out this same idea by going to the office in the morning, and charging these hard-earned fees, with no expectation of collecting them for weeks, months, years, and perhaps never.—Now how is it with these same dearly beloved patrons; will they bring in a fatted calf, two or three fattened hogs, a load of bacon, apples, corn, wheat, or even their eggs, butter, chickens, or turkeys, or anything else they have to sell, and distribute them about town on credit, to even the wealthiest men? No, indeed! They must have money or its equivalent for *their* effects. One might suppose from this state of things that the health and life of a human being are of less value than a yard of calico, a hickory shirt, a pair of stoga boots, a basket of eggs not above suspicion, or a skim milk cheese—whose inhabitants are sometimes more numerous than the city of Decatur.

Now, does not this fault, in a great measure, lie at our own doors? Most certainly, gentlemen. And what is the result of this imbecility on our part?—Poverty, embarrassment, and almost bankruptcy to the medical profession. It is not for want of skill and energy in our profession. I will venture to assert, without fear of contradiction, (leaving your humble speaker out of the question) that there cannot be found in any other place the size of Decatur, in the United States, more intelligence, skill, talent, and true moral worth in the profession. And it has been so for many years, and has been so regarded, not only by the citizens of Decatur and its vicinity, but by the State at large.

It is a fact well known to each of you, that the delegates from Macon County Medical Society exercise more influence in the State Association than those from any other, except the Chicago Medical Society.

In the early settlement of this county a tooth could hardly be extracted, or a finger amputated, by the ablest physician living in this section of country.—How does this question stand to-day? Is it necessary for any one of our citizens to leave home with all its endearments, to avail himself of the services of a POPE or a GROSS, in the hospitals of a strange city? Not by any means.—The most difficult operations in surgery, from the extirpation of a tumor, the amputation of limbs, the ligation of the deep-seated arteries, or the great operation of lithotomy can all be performed with as steady and firm a hand in this beautiful inland city, as in any other city in the Union.

Look at the appointments received by many of our physicians in the Union army. Every one of them receiving the place solicited, without a single rejection. Did one of them return home for want of skill, or on account of intemperance, or the want of ability on his part to discharge the duties of a physician and surgeon? No, my friends. Each returned home honored and respected alike by all.

Gentlemen, all that we ask is a fair remuneration for our services, as soon as practicable after services are rendered. This, I regret to say, has not been our good fortune in the past. Take, if you please, gentlemen, many of the physicians now living here, who have labored long and faithfully in this time-honored calling, for the people of this country, justly earning from forty to fifty thousand dollars during the last ten or fifteen years. And I ask you in the name of High Heaven what they have to show for it? This question I leave for you to answer. Now, my brothers, compare this state of things with the present condition of other men who came here about the same time, with but little capital, and engaged in other occupations, and what is the result? You find them building their brick and marble halls, living in fine residences, costly and elegantly furnished, their families not only enjoying all the necessaries and comforts, but all of the luxuries that wealth can purchase.

Now, we do not envy them in the enjoyment of all these things, but, on the contrary, rejoice that their efforts have been crowned with success, but believe it is high time that we, as a profession, should change our tactics, and may learn profitable lessons from those around us, in the successful prosecution of their various enterprises.

A reform should at once be established, and rigidly adhered to, in the collection of our just dues. If this were so, we would experience a happy change in our circumstances. The physician who is worthy of the name, with all the vast responsibilities of his profession resting upon his shoulders, should never experience any pecuniary embarrassment. He should never be compelled to submit to that humiliating spectacle of making himself a public beggar on the corners of the street, soliciting the amount due him from his friends. Instead of this he should be able to devote his whole time and skill to the cause of suffering humanity.

Now, gentlemen, so far as I am individually concerned, my mind is fully made up on this important question, and it would afford me much pleasure to act in concert with you all in this laudable effort to collect our just earnings.

So far as charity practice is concerned, I am not only willing to do my share, but will do it with the greatest of pleasure.

Gentlemen, language is wholly inadequate to express my feelings of gratitude and love for the extreme kindness and forbearance which you have manifested towards me, since I have had the honor of presiding during the deliberations of this Association, for the past year.

When I commenced addressing you, I did not design saying so much on this subject, but it is one upon which I have felt very deeply and sadly, and if I have wearied your patience, I ask your pardon.

